# Transcriptomic data for analyzing global gene expression patterns in Methicillin-resistance *Staphylococcus aureus* in response to spermine and oxacillin stress

**DOI:** 10.1016/j.dib.2018.11.090

**Published:** 2018-11-23

**Authors:** Shrikant Pawar, Xiangyu Yao, ChungDar Lu

**Affiliations:** a Department of Biology, Georgia State University, 33 Gilmer Street SE, 30303 Atlanta, GA, USA; b Department of Computer Science, Georgia State University, 33 Gilmer Street SE, 30303 Atlanta, GA, USA; c National Institutes of Health, 9000 Center Dr, 20892 Bethesda, MD, USA; d Department of Clinical Laboratory and Nutritional Sciences, Weed Hall 320, 01854-5125 Lowell, MA, USA

## Abstract

Methicillin-resistant *Staphylococcus aureus* (MRSA) is a rapidly emerging bacteria causing infection, which has developed resistance to most of the beta-lactam antibiotics because of newly acquired low-affinity penicillin binding protein (PBP2a), which can continue to build the cell wall when other PBPs are blocked by beta-lactams. Exogenous spermine exerts a dose dependent inhibition effect on the growth of *E. coli, Salmonella enterica serovar* and *Staphylococcus aureus*. We have selected an *MRSA Mu50* derivative which harbors mutation on *PBP2* gene (named as *MuM*) showing spermine resistance and which confers a complete abolishment of spermine-beta-lactam synergy. A transcriptomic profiling of *MuM* against *Mu50* (wild type) without any treatment, *MuM* and *Mu50* in response to high dose spermine and *Mu50* in response to spermine-beta-lactam synergy is provided in this article. These comparisons will enhance our current understanding of mechanisms of spermine-beta-lactam synergy sensitization effects on MRSA.

## Specifications table

**Table:** 

Subject area	*Bioinformatics*
More specific subject area	*Comparative genomics.*
Type of data	*Table, Figure, Microarray data*
How data was acquired	*The cDNA synthesis, fragmentation, and terminal labeling were carried out as per the protocols of the manufacturer (Affymetrix, Massachusetts, USA). Labelled cDNA was hybridized to the GeneChip Staphylococcal aureus genome array. After scanning, the images were processed with GCOS 1.4 software (Affymetrix, Massachusetts, USA).*
Data format	*Raw, analyzed.*
Experimental factors	*Treatment with Spermine, Oxacillin and in combination*
Experimental features	*The experimental features are compared between treatments MuM against Mu50 (wild type) without any treatment, MuM and Mu50 in response to high dose spermine and Mu50 in response to spermine-beta-lactam synergy.*
Data source location	Department of Biology, Georgia State University, 33 Gilmer Street SE, 30303 Atlanta, GA, USA
Data accessibility	Data is with this article. Also, the raw data files can be found at the GitHub repository by following the link: https://github.com/spawar2/Transcriptomic-data-for-analyzing-global-gene-expression-patterns-in-MRSA
Related research article	*Not applicable*

## Value of the data

• The data generated can be used to systematically compare *Mu50* (wild type) and *MuM* strains response to spermine alone (high dose) or in combination with b-lactam (oxacillin) (both at low dosages) using microarrays. A detailed transcriptomic analysis of how *MRSA Mu50* derivative harboring mutation on *PBP2* gene (named as *MuM*) showing spermine resistance responds to spermine and spermine-beta-lactam synergy was still unknown, this is the attempt to fulfill this gap.
• This data can be used to understand *Staphylococcus aureus* response to spermine and beta-lactams with mutated PBP2 protein. A strong relation between PBP2 protein and general stress *sigB* response, iron, potassium and polyamine transport systems was observed.
• The data can be used for future studies on the molecular mechanism of spermine interactions holding great potential for the development of new therapeutics for MRSA infections.

## 1 Data

**Fig. 1:**
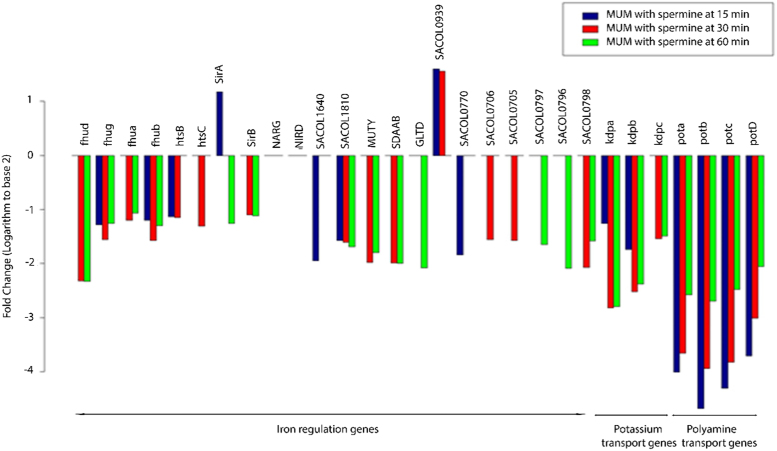
Bar graph with fold changes (logarithm to base 2) for iron regulation, potassium and polyamine transport genes in *MuM* strain at 15, 30 and 60-min time points with spermine treatment.

**Fig. 2:**
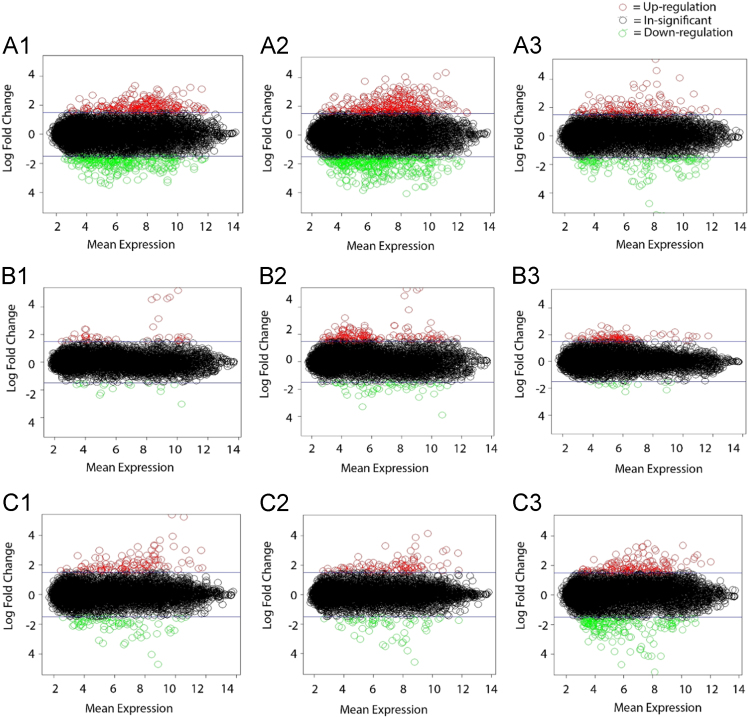
MA plots showing differentially expressed genes in *Mu50* and *MuM* treatments Fig. 2A.1, 2A.2 and 2A.3: *Mu50* at 15, 30 and 60-min time-points with spermine treatment over *Mu50* 0 min time-point without spermine treatment. Fig. 2B.1, 2B.2, 2B.3: MuM at 15, 30 and 60-min time-points with spermine treatment over *MuM* 0 min time-point without spermine treatment. Fig. 2C.1, 2C.2, 2 C.3: *MuM* at 15, 30 and 60-min time-points with spermine treatment over *Mu50* 0 min time-point without spermine treatment. Red color: Genes with greater than 1.5-fold change expression levels/up-regulated Green color: Genes with less than 1.5-fold change expression levels/down-regulated Black color: Insignificant expression levels Fig. 2A–C were generated with following ratios amongst treatments (/ sign is a ratio): A. *Mu50* 15-min time point spermine/*Mu50* 0 min time point without spermine B. *Mu50* 30-min time point spermine/*Mu50* 0 min time point without spermine C. *Mu50* 60-min time point spermine/*Mu50* 0 min time point without spermine D. *MuM* 15-min time point spermine/*MuM* 0 min time point without spermine E. *MuM* 30-min time point spermine/*MuM* 0 min time point without spermine F. *MuM* 60-min time point spermine/*MuM* 0 min time point without spermine G. *MuM* 15-min time point spermine/*Mu50* 0 min time point without spermine H. *MuM* 30-min time point spermine/*Mu50* 0 min time point without spermine I. *MuM* 60-min time point spermine/*Mu50* 0 min time point without spermine.

In first condition, *Mu50* and *MuM* strains were treated with 1 mM spermine and RNA was isolated at 0, 15, 30 and 60-min time-points with spermine and single 0 min time-point without spermine. In second condition, three treatments of *Mu50* strain with 1 mM spermine, 2 ng/μl oxacillin and a combination of 1 mM spermine, 2 ng/μl oxacillin were grown for one hour subsequently followed by RNA isolation. Labelled cDNA was hybridized to the GeneChip Staphylococcal aureus genome array. After scanning, the images were processed with GCOS 1.4 software (Affymetrix, Massachusetts, USA). The raw data files (.CEL) consist of intensity values of more than 10,000 genes with information of perfect and mis-match (PM and MM) probes. Each file is named according to the treatment and its generated time point. The normalization and analysis data is provided in respective Supplementary files with logarithm to base 2 fold changes. The raw files can be read in R using Bioconductor package *“Affy”* for replication and additions in analysis. List of iron regulation, polyamine and potassium transport genes with their significant fold change expression levels (logarithm to base 2) are provided in Table 1. Table 2 lists the plasmids used in this study. Bar graph with fold changes (logarithm to base 2) for iron regulation, potassium and polyamine transport genes in MuM strain at 15, 30 and 60-min time points with spermine treatment are shown on Fig. 1. MA plots showing differentially expressed genes in Mu50 and MuM treatments are provided in Fig. 2.

**Table 1:** List of iron regulation, polyamine and potassium transport genes with their significant fold change expression levels (logarithm to base 2). Comparisons with only genes that satisfy a significant *p*-value (less than 0.05) threshold are selected.

Gene symbol	Affymetrix ID	*MUM.NOSPM.0*	*MUM.SPM.15*	*MUM.SPM.30*	*MUM.SPM.60*
*fhud*	sa c914s711 at	1.39	0	−2.32	−2.33
*fhug*	sa c7993s6980 at	0	−1.28	−1.56	−1.26
*fhua*	sa c5423s4693 a at	0	0	−1.2	−1.07
*fhub*	sa c7989s6976 at	0	−1.2	−1.57	−1.3
*htsB*	sa c4643s3963 a at	0	−1.13	−1.15	0
*htsC*	sa c4639s3961 a at	0	0	−1.31	0
*sirA*	sa c1230s1008 at	0	1.18	0	−1.26
*sirB*	sa c1172s953 a at	0	0	−1.1	−1.12
*NARG*	sa c5574s4827 a at	−2.99	0	0	0
*NIRD*	sa c5580s4836 a at	−1.94	0	0	0
*SACOL1640*	sa c2711s2285 a at	0	−1.95	0	0
*SACOL1810*	sa c3357s2894 a at	0	−1.57	−1.61	−1.69
*MUTY*	sa c3689s3168 a at	0	0	−1.98	−1.8
*SDAAB*	sa c6092s5283 a at	0	0	−1.99	−2
*GLTD*	sa c7412s6438 a at	0	0	0	−2.08
*SACOL0939*	sa c8086s7067 a at	0	1.6	1.56	0
*SACOL0770*	sa c8202s7182 a at	0	−1.84	0	0
*SACOL0706*	sa c7993s6980 at	0	0	−1.56	0
*SACOL0705*	sa c7989s6976 at	0	0	−1.57	0
*SACOL0797*	sa c8283s7260 a at	0	0	0	−1.65
*SACOL0796*	sa c8276s7256 a at	0	0	0	−2.09
*SACOL0798*	sa c5353s4626 a at	0	0	−2.07	−1.58
*kdpa*	sa c4298s3650 a at	1.88	−1.26	−2.82	−2.8
*kdpb*	sa c4292s3644 a at	0	−1.74	−2.52	−2.38
*kdpc*	sa c236s9562 at	1.32	0	−1.54	−1.49
*pota*	sa c5349s4625 a at	−1.01	−4.01	−3.66	−2.58
*potb*	sa c9028s7925 a at	−1.97	−4.68	−3.94	−2.7
*potc*	sa c795s596 a at	−1.36	−4.31	−3.83	−2.48
*potD*	sa c803s604 a at	−1.69	−3.71	−3.01	−2.06

**Table 2:** Plasmids used in this study.

Plasmids	Relevant characteristics	Source or reference
*pBAD/HisA*	Expression vector, Amp	Invitrogen
*pBAD/HisD*	Expression vector for producing N terminal His tag fusion, Amp	This study
*pBAD/HisE*	Expression vector for producing C terminal His fusion, Amp	This study
*pH6N-PBP1*	*pBAD/HisD* expressing N-His-PBP1	This study
*pH6C-PBP2*	*pBAD/HisE* expressing C-His-PBP2	This study
*pH6N-PBP3*	*pBAD/HisD* expressing N-His-PBP3	This study
*pH6N-PBP4*	*pBAD/HisD* expressing N-His-PBP4	This study

## 2 Experimental design, materials and methods

### 2.1 Bacterial strains, plasmids, and growth conditions

*Staphylococcus aureus Mu50*, RN4220 and *Escherichia coli DH5 alpha* were used for this study. With oxacillin and spermine MIC׳s of 512 μg/ml and 1 mM (pH 8.0), spontaneous mutants of MRSA *Mu50* were obtained by spreading 1 × 108 colony forming units (CFU) of log-phase cells on spermine-containing plates with Luria-Bertani (LB) medium (37 °C overnight). One colony found resistant to spermine was labelled as *MuM.*

Protein cloning, purification and expression: Genes *pbp1*, *pbp2, pbp3*, and *pbp4* were amplified without N-terminal signal peptide and the transmembrane domain from *Mu50* strain. Generated plasmids were then cloned (*PstI/EcoRI* restriction sites) into *pBAD/HisD* vector with a hexahistidine tag. Recombinant proteins were then expressed from these plasmids. Proteins PotD and PotR of the *potABCD* operon were expressed in similar way. Plasmids were expressed in *Top10 E. coli* strains (30 °C) in LB medium supplemented with arabinose (0.2%). Proteins bound on HisTrap HP column (GE) were eluted by imidazole (500 mM).

### 2.2 Complementation of pbpB

The *pbpB* gene is transcribed independently or from its upstream *prfA* promotor as a polycistronic RNA [1]. Using a shuttle vector *pCN38* the PCR product was cloned into the *BamHI* and *NarI* sites. Plasmid DNA isolated from strains *RN4220* was introduced into *Mu50* and *MuM* strains by electroporation.

Transcriptional profiling conditions: *Staphylococcus aureus Mu50* and *MuM* were grown in Tris-buffered LB (pH 7.5), and treated with the RNA protection reagent followed by harvestation. RNA was isolated at 0, 15, 30 and 60-min time-points with spermine (1 mM) and 0 min time-point without spermine. For oxacillin stress analysis, *Mu50* strain was exposed with spermine (1 mM), oxacillin (2 ng/μl) and combinations (1 mM and 2 ng/μl of spermine and oxacillin). We found that the spermine (0.5 mM) can stimulate oxacillin MIC from concentrations 512 μg/ml to 1 μg/ml, so we chose to use 1/4 MIC instead of 1 mM for spermine, and 1/32 MIC instead of 16 μg/ml for oxacillin [2]. Extraction of RNA samples was performed using phenol and digestion with RNase-free DNase I for removing genomic DNA. The Affymetrix GeneChip *Staphylococcal aureus* genome array chips requires its specific protocols for cDNA synthesis, fragmentation, and terminal labeling, which was followed accordingly for all the samples. The GCOS 1.4 software was used to process images after scanning, and the data was generated for two independent biological replicates.

Microarray analysisMas 5.0 normalization was performed for all the files at 0-min time point for Mu50 and MuM strains and strains with MuM–PBP2 and Mu50–PBP2 complementation plasmid [3]. For calculating upregulated genes, in control of all the P (present) call intensity values were considered for analysis and all the *M* (marginal) and *A* (absent) calls were regarded as 100. For treatment, all the genes with intensity values above 500 were considered in the analysis. This rigorous approach gave us significant differences amongst various comparisons avoiding any false positive and false negative results. The exact opposite criteria were applied to find down-regulated genes. Fold changes (> 1.5 and < 1.5) with MuM–Mu50 and (MuM with PBP2)–(Mu50 with PBP2) were taken to find up and down-regulated genes in MuM strain [4]. A similar method was used for comparison of MuM and Mu50 at 15, 30 and 60-min time points with spermine treatment. Up and down-regulated genes were calculated and compared with 0-min time point with no spermine treatment. All the microarray data were analyzed using library ‘Affy’ package [5] on R platform [6]. Heat maps were generated using library ‘gplots’ [7]. Heat maps were developed on Z scores, which were calculated by heatmap.2 function of gplots [*Z* score = (raw intensity − average)/standard deviation]. MA plots [8] for showing differentially expressed genes were calculated as follows: *M* = Logarithm to base 2 (Treatment/Control), *A* = 1/2 × Logarithm to base2 (Treatment × Control). MA plots were made on R platform with ‘plotMA’ limma Bioconductor package [9], [10], [11], [12].
